# Monte Carlo verification of radiotherapy treatments with CloudMC

**DOI:** 10.1186/s13014-018-1051-9

**Published:** 2018-06-27

**Authors:** Hector Miras, Rubén Jiménez, Álvaro Perales, José Antonio Terrón, Alejandro Bertolet, Antonio Ortiz, José Macías

**Affiliations:** 1 Department of Medical Physics, Hospital Universitario Virgen Macarena, Av. Doctor Fedriani 3, 41009 Seville, Spain; 2 Biomedicine Institute of Seville (IBiS), Antonio Maura Montaner, 41013 Seville, Spain; 3 R&D Division, Icinetic TIC SL, Av. Eduardo Dato 69, 41005 Seville, Spain; 4 Atomic, Molecular and Nuclear Physics Department, Universidad de Sevilla, Av. Reina Mercedes s/n, 41012 Seville, Spain

**Keywords:** Cloud computing, Monte Carlo, Radiotherapy

## Abstract

**Background:**

A new implementation has been made on CloudMC, a cloud-based platform presented in a previous work, in order to provide services for radiotherapy treatment verification by means of Monte Carlo in a fast, easy and economical way. A description of the architecture of the application and the new developments implemented is presented together with the results of the tests carried out to validate its performance.

**Methods:**

CloudMC has been developed over Microsoft Azure cloud. It is based on a map/reduce implementation for Monte Carlo calculations distribution over a dynamic cluster of virtual machines in order to reduce calculation time. CloudMC has been updated with new methods to read and process the information related to radiotherapy treatment verification: CT image set, treatment plan, structures and dose distribution files in DICOM format. Some tests have been designed in order to determine, for the different tasks, the most suitable type of virtual machines from those available in Azure. Finally, the performance of Monte Carlo verification in CloudMC is studied through three real cases that involve different treatment techniques, linac models and Monte Carlo codes.

**Results:**

Considering computational and economic factors, D1_v2 and G1 virtual machines were selected as the default type for the Worker Roles and the Reducer Role respectively. Calculation times up to 33 min and costs of 16 € were achieved for the verification cases presented when a statistical uncertainty below 2% (2σ) was required. The costs were reduced to 3–6 € when uncertainty requirements are relaxed to 4%.

**Conclusions:**

Advantages like high computational power, scalability, easy access and pay-per-usage model, make Monte Carlo cloud-based solutions, like the one presented in this work, an important step forward to solve the long-lived problem of truly introducing the Monte Carlo algorithms in the daily routine of the radiotherapy planning process.

## Background

Monte Carlo (MC) simulations have become the gold standard for dose calculation in radiation therapy treatments since they include the real physical processes involved in the interaction of photons with matter in general and human tissues in particular [1, 2]. Some of the codes most frequently used in the radiation therapy field are, for example, EGSnrc [3], MCNP [4], PENELOPE [5] or GEANT4 [6]. Making use of the named codes, some friendly-user software is often developed. For example, for PENELOPE code, PenEasy, a general-purpose main program [7], and PRIMO, an application for clinical linacs MC calculations with graphical user interface included [8], are available.

Regardless of the code used, a huge number of simulated particles is necessary to achieve a precise solution because of the stochastic nature of the MC approach. Therefore, these simulations are often computationally-expensive or time-consuming [9]. A possible approach to handle this is the use of cluster-based parallel computing for speeding up MC simulations [10]. The main barrier to this solution is the high investment needed, as well as associated maintenance, upgrade and staff costs [11]. Such costs make practically unfeasible the use of MC simulations in a routine clinical basis.

Another proposed way to address the MC computational cost is the use of the graphics processing unit (GPU), whose architecture seems suitable for parallel computations since it comprises thousands of processing units on a single chip [12–14]. However, the size of the memory of this kind of devices is very limited compared to CPU-based implementations. This, together with other issues, makes their performance worse than what it could be expected as it was shown in a recently published point-counterpoint [15].

A more economically efficient approach is the use of the Cloud, which essentially consists of a set of computing resources offered through internet as a pay-per-usage service [16]. In a Cloud Computing environment it is easy to create a virtual cluster with the capability of distributing any tasks onto the multiple computing nodes, which makes parallel computation available. Using such an approach, there is no need for initial investment since the facilities are already built and their maintenance is assumed by the owning companies. Instead, the whole outlay is about the costs of the resources actually used. Furthermore, applications can be scalable, so their computational resources can change at runtime to match the real needs, while the capacity of a conventional cluster is fixed, so the efficiency might not be optimal [17]. The likelihood of future implementation of the Cloud Computing paradigm in the routine of clinical radiation therapy has been highlighted [18].

In a previous work [19], we presented CloudMC, a cloud-based platform developed over Microsoft Azure® cloud. It was originally intended to provide computational power to run MC simulations in a short time. This is accomplished through the distribution of the calculations over a dynamic cluster of virtual machines (VMs) that are provisioned on demand and removed automatically once the simulation is finished.

CloudMC was designed following some basic premises:

- Accessibility: As CloudMC is presented as a web application, it is accessible to any user through internet, without the need of installing any software or acquiring any hardware.
- Multi-application: It is possible to run different MC programs independently on the MC code in which they are based on.
- Non-intrusiveness: There is no need of modifying the code or the MC programs in order to be executed on this platform.
- Elasticity: The computational resources are not fixed, the user is able to select the number of computational nodes in which the calculations will be distributed.

During the last years, new developments have been implemented on CloudMC to include the service of MC verification of radiotherapy (RT) treatments and to improve its efficiency. This developments are presented in this work, together with a study of the performance of CloudMC for MC verification service.

## Methods

### CloudMC

**Fig. 1:**
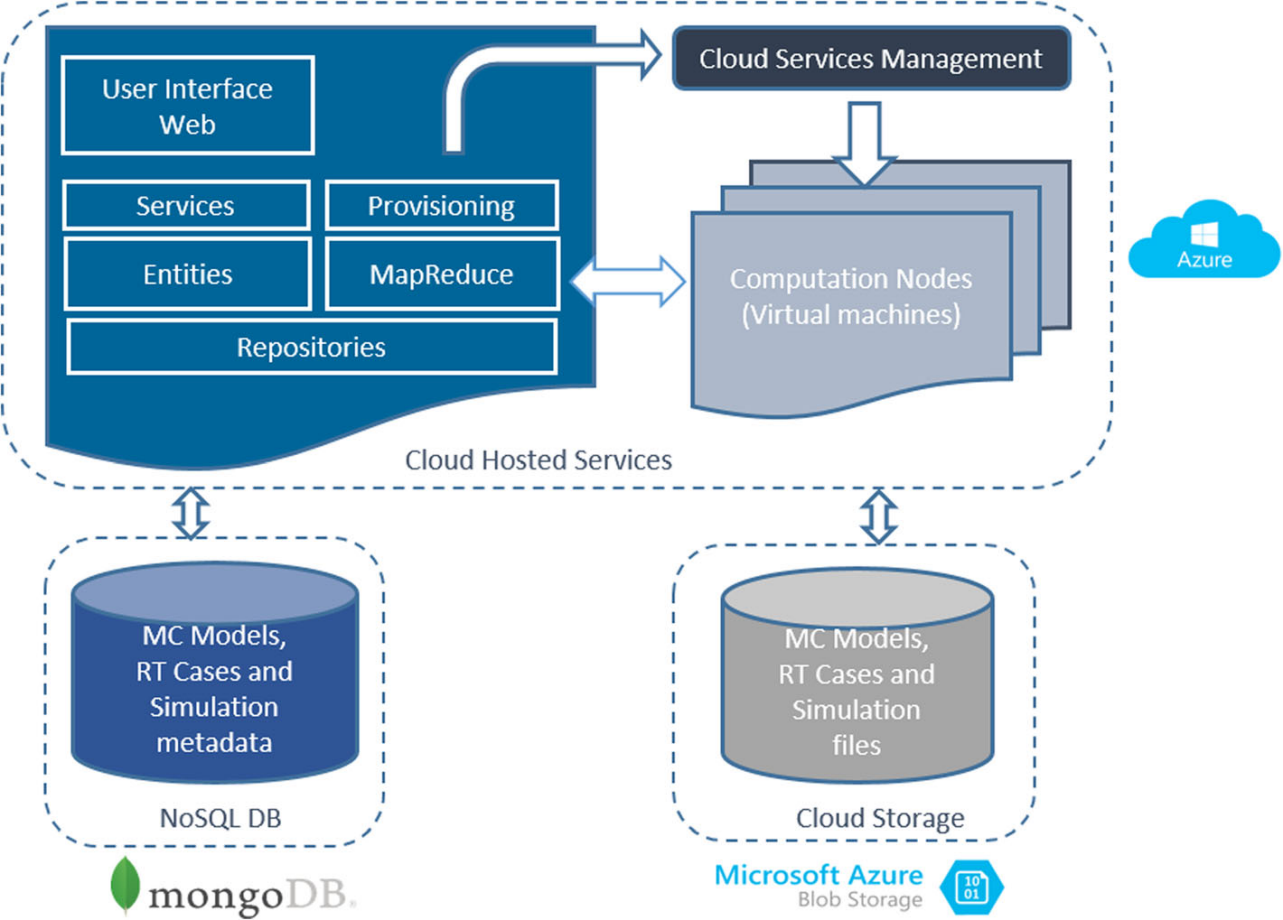
CloudMC architecture

CloudMC architecture is shown in Fig. 1. From the software architectural point of view, CloudMC follows a classical n-layered architecture, making wide use of dependency injection across the different layers in order to loose coupling. This means that the software is composed by several autonomous modules, allowing an easier testing and evolution of the whole system.

The first layer is the user interface, which in CloudMC is a web application. As such, it only requires a web browser to be used. It is based on a Microsoft web framework called Microsoft ASP.Net MVC 4. It follows, therefore, a model view controller architecture, a widely used pattern in the development of software user interfaces that break down them in three components: model, view and controller. In CloudMC, views are mainly HTML pages with some Razor [20] components and Javascript utilities to improve interactivity and user experience. Controllers are C# [21] (a type-safe object-oriented programming language) classes supporting, mainly, typical CRUD operations (Create, Read, Update and Delete) for the entities CloudMC manages.

**Fig. 2:**
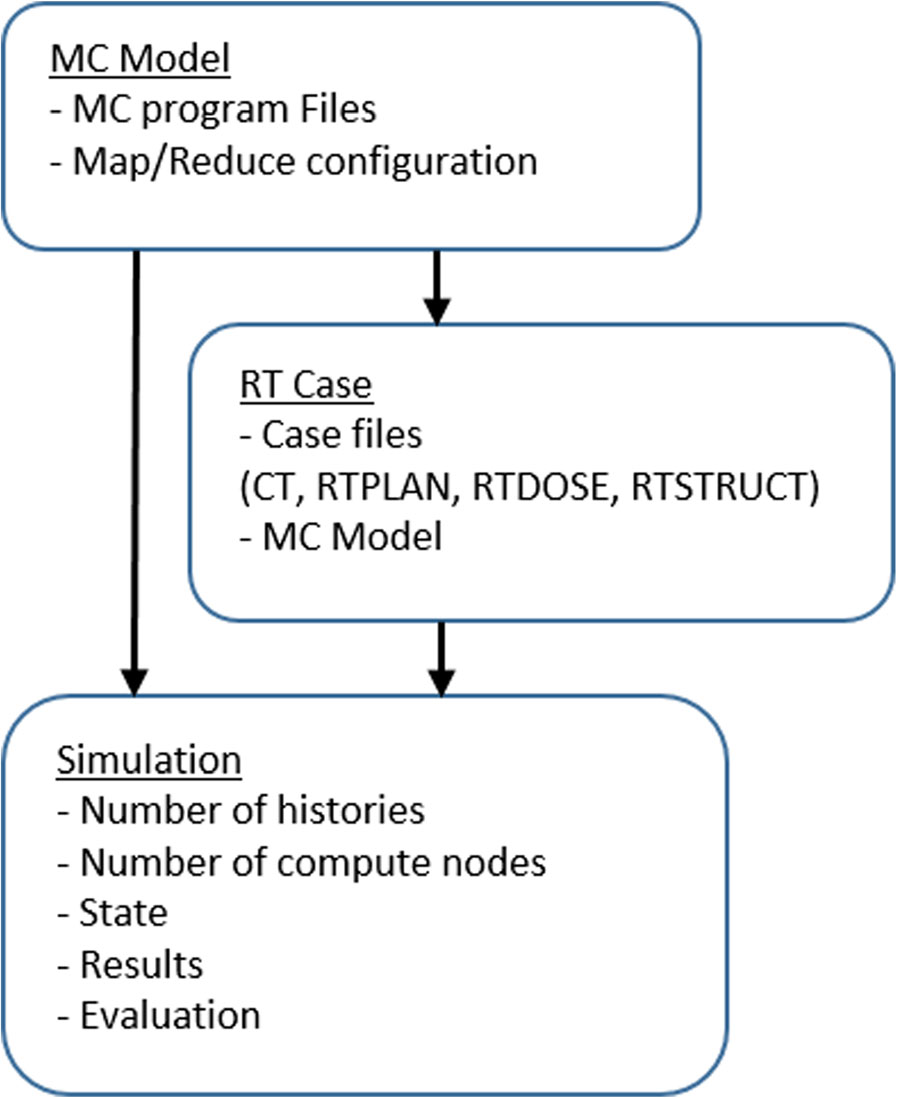
Representation of the main CloudMC entities

On the center of this architecture, there is an *Entities* layer [22], where key domain concepts are defined as plain C# classes. Figure 2 is a diagram of the three main entities and their dependencies. The main concept is the *MC Model* entity, which represents a group of files that can run a MC simulation. Associated to this *MC Model*, there are several entities that represent the configuration of how to run this *MC Model* in parallel. Basically, they determine the files and the position, inside of these files, where the input parameters (number of histories, execution time or random seeds and the mobile geometric elements) that have to be modified are located as well as the contents of the output files and their formats.

The second important entity is the *RT Case*. This entity contains the set of patient specific files that define a RT treatment and an instance of the *MC Model* that will be used to perform the MC calculations.

The third important entity is the *Simulation*. A *Simulation* can represent an execution of either a *MC Model* or a *RT Case*. A *Simulation* is also configured with the number of execution nodes that will be used for the parallelization, and with other parameters like number of histories to simulate. *Simulation* has a state (Inactive, downloading files, simulating, uploading results and finished) and, in case it is finished, a list of output results and an evaluation.

The following layer is *Services*. It contains several C# services that coordinates all the steps to create a simulation, to run it in parallel on the computation nodes, and to collect the results once the simulation is finished. *Services* layer uses the *MapReduce* module to configure *MC Model* files for parallelization, based on *MC Model* instance configuration. *MapReduce* module contains the cornerstone of the logic of CloudMC, which allows to achieve the parallel execution of different types of Monte Carlo applications (map) and merge their results (reduce).

Furthermore, *Services* layer uses the *Provisioning* module to create the compute nodes that will run the simulation. Currently, CloudMC relies on Microsoft Azure. Thus, the *Provisioning* module communicates with Azure Services Management REST API, a Microsoft Azure interface that relies on HTTP protocol which allows other software systems to consume its services, to request the creation/deletion of the compute nodes.

Finally, the *Repositories* layer handles the persistence of the entities and assets of CloudMC. Two types of persistency technologies are used; on one hand, there is a document database [23], specifically MongoDB, that stores entities metadata. On the other hand, all the files corresponding to *MC Model*, *RT Case* and *Simulation* are stored in Microsoft Azure Storage, a cloud object storage for unstructured data.

The new features added to this version of CloudMC are:

- ○ Implementation of Evil-DICOM library [24], a C# class library for reading and manipulating DICOM files [25].
- ○ PlanRT class for reading RT plans in DICOM format exported from a treatment planning system (TPS). It contains methods to transform different types of dynamic beams into a discrete set of static beams that can be calculated by MC simulation.
- ○ CTimage class for reading the patient CT image set and convert it to PENELOPE (PenVox) or EGS (egsphan) voxelized geometries from a HU / density-material conversion table defined by the user. This conversion has also the possibility to change the size and resolution of the voxelized phantom.
- ○ RTDose class. It allows to read, modify and write dose distribution files in DICOM format (RTDOSE). The final dose distribution of the MC verification is transformed into this format to facilitate the evaluation with other programs.
- ○ PlanEval is a set of classes that makes it possible to read dose matrix and structure files in DICOM format (RTDOSE and RTSTRUCT) for treatment evaluation purposes. For instance, calculating dose-volume histograms (DVH).
- ○ Geometry Mapper is a set of methods that are used to manage the information related to mobile geometric elements (isocenter shifts, gantry, collimator and table angles, MLC and jaws positions, etc.) contained in the MC input files. These files are modified for each verification case with the information read from the corresponding DICOM RT plan. It also has a method to distribute the calculations of the treatment beams over the available computing nodes. Two options are implemented: “Equal” and “MUWeighted”. With the “Equal” option the different beams are distributed in the same number of computing nodes, while with the “MUWeighted” option the beams are distributed in a number of nodes proportional to their weight in terms of relative MU.
- ○ Reducing binary outputs. In the first versions of CloudMC, the output could only be managed if it was a text file with data in column format. A parametrization for reducing general binary files has been implemented. These files are supposed to have a header, which will not be modified in the reducing process, and the dose distribution data followed by the corresponding uncertainty distribution in single or double precision format.

### Azure roles tests

In Azure, a Cloud Service Role is a collection of managed, load-balanced, Platform-as-a-Service VMs that work together to perform common tasks. There are two varieties: Web Role and Worker Role. A Web Role is a Cloud Service role where web applications are implemented. These applications contain the user interface through which the user interacts with and are developed through programming languages / technologies that are supported by Internet Information Services (IIS), such as ASP.NET, PHP, Windows Communication Foundation and Fast CGI. A Worker Role is any VM in Azure that runs applications and services level tasks. They are mainly used to perform supporting background processes along with Web Roles. Worker Roles can only be developed with .NET.

The VMs that support Azure roles can be of different types and sizes [26]. VMs are classified in several series. The ones considered in this work are described below.

- A-series: General purpose VMs. Can be deployed on various types of hardware and processors. They were the only type of machines eligible during the early times of Azure.
- D-series: Optimized compute. D-series VMs are designed to run applications that demand higher compute power and temporary disk performance. D-series VMs feature a solid-state drive (SSD), faster processors and a higher memory-to-core ratio than A-series.
- Dv2 and Dv3-series: Next generation of D-series. Their CPU is about 35% faster than the D-series CPU. They are based on the latest generation 2.4 GHz Intel Xeon® E5–2673 v3 (Haswell) processor and with Intel Turbo Boost Technology 2.0 that can go up to 3.2 GHz.
- Ev3-series: Memory intensive VMs. Running on the Intel® Broadwell E5–2673 v4 2.3GHz processor, and the Intel® Haswell 2.4 GHz E5–2673 v3.
- G-series: Memory optimized and high memory-to-core ratio VMs that feature Intel® Xeon® processor E5 v3 family.

Inside each series, it is possible to choose between several sizes of VMs, i.e. different amount of cores, RAM, temporary storage, etc. The price-per-hour of a VM depends on its type and size.

Some tests were conducted in order to determine the most suitable type and size for the set of Worker Roles that run the MC simulations in CloudMC and for the role responsible for the reducing tasks, the so called Reducer Role in this paper. For performance benchmarking of the different types and sizes of Worker Roles, a PenEasy [7] execution corresponding to a 3·10^5^ histories MC simulation of an iodine radioactive seed in a COMS ophthalmic applicator [27] has been run on a single machine of different type/size each time. The tally files resulting from the PenEasy simulations contain the information of the spent CPU time, which will be used to evaluate the efficiency of the different VM types in executing this task.

The test for the Reducer Role consisted in the execution of a MC simulation of a radiotherapy beam on a patient phantom in 500 Worker Roles. Then, different types of VMs were used to perform the reduce tasks of the output files generated by the Worker Roles. Each Worker Role produces two output files, a 12 MB binary dose distribution file and an 8 MB IAEA PHSP. The time spent on the reduce task and on uploading the final results to the storage for each Reducer type was evaluated.

### MC treatment verification in CloudMC

In order to show the performance of the MC treatment verification process on CloudMC, three different cases have been selected corresponding to three different treatments in three different LINAC models and involving the use of three different MC codes. The aim of considering so many variables was to prove that the conclusions are applicable to a wide range of cases. All these cases have the same structure in common. The calculations start from a source phase space file (PHSP), in IAEA format [28], previously calculated at the plane just before the beam modifiers (jaws and MLC). This source PHSP is used by the MC program that contains the MC model of the linac to generate the secondary PHSPs at the end of the beam modifiers. Finally, these secondary PHSPs are used as source by PenEasy to obtain the dose distribution inside a voxelized geometry built from the CT image set of the patient.

The previous calculation of the source PHSPs has also been performed with CloudMC using the corresponding MC Model for each linac. The generated PHSPs contain more than 5·10^8^ particles and are larger than 15 GB.

Case 1: mArc H&N treatment planned for a Siemens ONCOR® LINAC with the 160-MLC multi-leaf collimator. The MC model uses an in-house developed program [29] based on the Geant4 code [6, 30, 31]. The mArc [32, 33] technique is the approach to volumetric therapy proposed by Siemens. It consists in a rotational beam divided in small arclets (of 2–4 degrees width), which in our case are 8 degrees apart from each other. While the gantry rotates, the beam is switched on only when the angle position is within the arclets. From the end of one arclet to the beginning of the next one the beam is switched off and the MLC moves to reach the next control point configuration. CloudMC reads the DICOM RT plan and transforms each arclet to a static beam with a gantry angle equal to the arclet central angle and the same MU delivered during the arclet. The voxelized phantom generated had a 2x2x5 mm^3^ resolution.

Case 2: Static IMRT prostate treatment planned for a Siemens PRIMUS® linac. The MC model used for this linac was developed by Leal et al. [34, 35] using the program BEAMnrc [36], based on the EGSnrc code [3]. The treatment consists of 25 control points distributed in seven incidences. The calculation voxel size was also 2x2x5 mm^3^.

Case 3: SBRT lung treatment planned for a Varian Clinac 2300® with 120-MLC. The back-end programs of PRIMO software [8], version 0.1.5.1307, were used for MC calculations (PenEasyLinac.exe and PenEasy_PRIMO.exe). The treatment consists of nine static beams conformed to the planning target volume (PTV). A smaller voxel size of 2.5 × 2.5 × 2 mm^3^ was used to match, like in cases 1 and 2, the same calculation grid resolution used in the TPS.

From these three RT cases, several simulations have been run changing the number of histories and the number of Worker Roles to study the feasibility of CloudMC to perform MC verification of RT treatments. The VM type chosen for the Worker Roles was the D1_v2, while a G1 VM was used for Reducer Role. For each case, two simulations were run using different number of histories in order to obtain results with two levels of uncertainty, one below 4% and another below 2% (2σ).

### PRIMO implementation in CloudMC

The PRIMO implementation in CloudMC has a special interest, because it allows to simulate in CloudMC all the LINACS modelled in the PRIMO software. In order to understand how it was implemented, we first need to present a brief explanation of PRIMO software. PRIMO is a MC platform that allows for the simulation of a wide variety of Varian and Elekta linacs. It makes use of the physics from PENELOPE code through the main simulation program PenEasy [7]. Dedicated variance reduction techniques have been implemented to reduce computation times. The main program PRIMO.exe contains the graphical interface through which the user configures the simulation as well as analyze the results. This program is also responsible for managing the back-end programs preparing their input, controlling the execution and collecting as well as presenting their results. These programs are PenEasy_PRIMO and PenEasyLinac. PenEasy_PRIMO is a dedicated version of the PenEasy code, while PenEasyLinac is a program that prepares the linac geometry and the input files for PenEasy.

When a PRIMO user launches a simulation, PRIMO transcribes the information defined by the user through the graphical interface into input text files for PenEasyLinac. Then PRIMO calls the execution of PenEasyLinac, which generates the input files for PenEasy_PRIMO. These input files consist of a main PenEasy input file, the material files and the linac geometry modified with the user defined beam configuration. PRIMO calls then the execution of PenEasy_PRIMO that carries out the MC simulation and manages the map/reduce tasks if the user had selected the parallelization in several cores.

PRIMO cannot be implemented as it is in CloudMC because CloudMC only works with programs that have text files as input and this is not the case of PRIMO. However, it is actually the case of its back-end programs. Subsequently, to create the MC model of PRIMO in CloudMC the input files of PenEasyLinac are parametrized for the map tasks. The file PenEasyLinPlus.in contains the information about the number of histories and the initial random seeds while the file PRIMOPEL.in contains the linac model name and the geometric configuration of the beam. The files required to create the MC model in CloudMC are mainly the ones contained in the PenEasyLinac folder, so this folder was uploaded completely into the corresponding container in the Azure Storage system.

Once this MC model of PRIMO is created in CloudMC it is possible to use all the features of the platform like performing MC verification of all sort of RT treatments calculated for any of the linac models contained in PRIMO.

## Results

### Virtual machine type tests

The results of the Worker and Reducer Roles performance tests in the different types of VMs are shown in Table 1. Information about the VMs specifications is also provided [26]. The outcome considered for the Worker Role test was the CPU time spent on the execution of the PenEasy MC program. CPU times are also presented relative to the A1 (Small) size (Rel. Time column in Table 1). The fastest machine was found to be the G1, but it is also the most expensive. It can be seen that the number of cores is not a factor to take into account for the calculation speed. The most influencing factor is the processor features. As it was previously explained, D-series are compute-optimized machines with faster processors than the A-series. Furthermore, Dv2-series are even faster, as they are based on the latest generation 2.4 GHz Intel Xeon® E5–2673 v3 (Haswell) processor.

**Table 1:** Characteristics of the different VM types and sizes (columns 2–5) and results of the execution speed test (columns 6–8) and the reducer test (columns 9 and 10)

VM type	CPU cores	RAM (GB)	Disk (GB)	Cost (€/h)	CPU time (min)	Rel. Time	Cost. Eff.	Merging time (min)	Upload time (min)
Small (A1)	1	1.75	225	0.0675	11.30	1.00	1.00	18.9	6.7
Medium (A2)	2	3.5	490	0.135	11.60	1.03	2.05	15.4	6.7
Large (A3)	4	7	1000	0.2699	11.87	1.05	4.20	11.5	6.7
D1	1	3.5	50	0.1249	7.32	0.65	1.20	19.8	5.2
D1_v2	1	3.5	50	0.1249	5.47	0.48	**0.90**	18.1	7.0
D2_v2	2	7	100	0.2505	5.08	0.45	1.67	8.3	7.0
D2_v3	2	8	16	0.1788	5.65	0.50	1.32	11.2	6.3
D3_v2	4	14	200	0.5001	5.48	0.48	3.59	3.6	6.4
D4_v2	8	28	400	1.001	5.09	0.45	6.68	3.9	4.0
D11	2	14	100	0.291	7.58	0.67	2.89	6.1	5.3
D11_v2	2	14	100	0.291	5.53	0.49	2.11	4.3	5.6
E2_v3	2	16	32	0.2126	5.65	0.50	1.57	4.1	5.5
E4_v3	4	32	64	0.4251	5.70	0.50	3.18	4.0	4.0
G1	2	28	384	0.6496	262	0.39	3.72	3.4	3.9
G2	4	56	768	1.2987	293	0.43	8.31	3.3	3.6

Most optimal cost-efficiency value highlighted in bold

The “cost-efficiency” factor, presented in the 8th column of Table 1, is calculated as the product of the time and the cost relative to the A1 machine. The VM type with best cost-efficiency is the D1_v2; this means that a simulation executed on this machine will cost less than the same simulation executed on any other of the machines analyzed.

To evaluate the performance of different types of VMs for the reduce tasks, the time spent by the Reducer Role on merging the simulation output files and uploading the final results to the Storage are presented in the lasts columns of Table 1. Two output files per Worker Role were generated in each simulation, an 8 MB IAEA phase space and a 12 MB dose distribution in binary format. Since the number of Worker Roles was set to 500, it means that the Reducer Role has to download and process 1000 files corresponding to 9.4 GB of data. The size of the reduced files that are finally uploaded to the storage is 3.77 GB. The time that the Reducer Role spends downloading the results of the Workers from the Storage is not considered. That is because the Reducer is already alive when the Workers are running the simulation and it is downloading the results in real time as the Workers are finishing.

VM types with high RAM have a similar performance for the reduce tasks. In order to choose one type as default, other features, like the disk capacity and the cost, need to be taken into account. For example, E-series machines have a good performance, but they have less disk capacity, which may not be enough for some simulations involving very large PHSPs. According to all this, G1 has been chosen as the preferred VM for the Reducer Role.

### MC verification cases

The results of the performance of three MC verification cases in CloudMC are presented in Table 2. For each case, two simulations were run. Simulation 1 produces a dose distribution with a 2σ uncertainty of around 4% in the PTV, while for simulation 2, a four times higher number of histories was selected to obtain a lower uncertainty, below 2% in the PTV. The Workers mean time is given with its associated standard deviation. The total simulation time reported corresponds to the interval since the user clicks the run button until the Reducer uploads the final results to the Storage. It includes the time needed to mount the Workers and Reducer cloud services, the start-up time (SUT) of the VMs, the execution tasks performed by the Workers and the processing tasks performed by the Reducer. The time required to upload the patient data to the application was not considered.

**Table 2:** Performance results of three different MC verification cases in CloudMC. For each case, the results of two simulations with different number of histories are presented

	Case 1		Case 2		Case 3	
MC programs	Geant4 + PenEasy		BEAMnrc + PenEasy		PenEasy_PRIMO	
LINAC	ONCOR		PRIMUS		CLINAC 2300	
RT treatment	H&N mArc		Prostate static IMRT		Lung SBRT	
Simulation #	1	2	1	2	1	2
Number of Workers	200	400	200	400	200	400
1st Worker SUT	8.1	5.6	6.4	7.9	5.9	5.9
Last Worker SUT	10.7	9.7	8.8	9.9	12.8	11.5
Workers mean time	6.8 ± 0.9	12.0 ± 1.9	3.5 ± 0.7	5.5 ± 1.4	7.7 ± 0.4	11.4 ± 0.8
Reducer merge time	1.3	2.4	1	1.9	3.1	8.3
Reducer upload time	1.2	1.2	0.4	0.7	0.9	1.25
Total simulation time	21.1	28.3	14.5	20.2	23.2	33.4
Uncertainty (k = 2)	3.6%	1.7%	2.9%	1.4%	3.8%	1.9%
Estimated cost (€)	4.3	15.7	2.6	7.4	5.5	13.6

*SUT* Start-up Time. All time measurements are given in minutes

From the two values of Workers mean time obtained for each case it is possible to estimate the non-parallelizable time. Non-parallelizable tasks are the ones that cannot be divided and, therefore, have to be done in all the Worker Roles: downloading files from the storage, initializing the MC programs, processing output files and uploading results to Storage. The non-parallelizable time is the main factor responsible for the increase in the cost when more compute nodes are used for parallelization. This time was estimated in 1.5, 1.6 and 3.9 min for the three cases presented respectively. The main reason for case 3 having larger non-parallelizable time is the smaller voxel size used. The CT data set is also larger than the ones in case 1 and 2 because the scan covers a larger anatomical region. This requires the manipulation of large files during all the simulation process and, consequently, it results in an increase of the time of the non-parallelizable tasks. This is also the reason for the larger merging times in the reducer phase.

## Discussion

The calculation speed is not the only feature to consider when choosing the most adequate VM type because the calculation times are reduced in CloudMC mainly by means of the parallelization strategy. The cost per hour is another important factor. The VM with best cost efficiency was shown to be the D1_v2 and, for this reason, it was the default type chosen for the Worker Roles in CloudMC.

When it comes to choosing the VM as the Reducer Role, its cost is not such an important factor because it will only contribute to a small proportion of the total cost of the simulation. The cost of a simulation is calculated from the usage time of every VM (Web Role, Worker Roles and Reduccer Role) and their cost per hour. Therefore, the main contribution to the simulation cost will come from the Worker Roles when a large number of them is selected. For all these reasons, having short reducing times was prioritized and the G1 is the default size for the Reducer Role in CloudMC.

One of the steps that increases the most the time of a simulation in CloudMC is the Worker Roles SUT. When a Worker Roles service is created in Azure, VMs have to be created over physical hardware and the operating system needs to be initialized. This may take some minutes. In Table 2 the SUT of the first and last Worker Roles is presented. It seems that there is no correlation between the number of Workers and the SUT of the first one or the time interval between the first and the last Worker initialized. The SUT contribution to the total simulation time might be removed if the Worker Roles service was already created before starting the simulation, but it would increase the final cost considerably.

The costs associated to MC verifications like those presented above have a strong dependency on several factors like the efficiency of the MC engine, the uncertainty level desired, the features of the VMs used, etc. It is important to point out that, since the release of the first commercial clouds, important upgrades have been made to provide more types of VMs optimized to perform different tasks, at the same time that the costs have been decreasing more and more. For example, at the time we published our previous work [19] in 2013, a little variety of VMs sizes was available and their cost was almost double as of today.

The results presented for the MC verification cases should not be understood as a comparison of the efficiency between different codes. There are many factors that influence the calculation times, like the simulation parameters (cutoff energies, variance reduction techniques…), the dimensions and voxel size of the patient voxelized phantom, etc. A more detailed study of the effect of these factors on the total simulation time could be done in order to minimize times and costs, but it is beyond the purpose of this work. Therefore, the aim of using different MC codes was not to make a comparison between them, but to show the flexibility of CloudMC.

In contrast to other initiatives that developed a highly integrated solution pursuing near real-time MC calculations in a TPS [37], CloudMC has been designed as a flexible platform independent of any commercial planning software that, at the same time, allows for the possibility of experimenting with different MC engines independently of the code they are based on.

Regarding the implementation of PRIMO in CloudMC, a new version of the PRIMO (version 0.3.1) has been recently released that incorporates new features and substantial changes like, for example, the possibility of using the fast MC code DPM [38] as the backend program to run the simulations. The implementation of the new PRIMO version in CloudMC has not been addressed yet, but it is part of our project roadmap.

## Conclusions

Following the path started in our previous work [19], the MC verification of RT treatments has been implemented in CloudMC. MC cloud-based solutions like the one presented here overcome the main drawbacks historically associated to the use of MC algorithms in clinical routine, as they take the main advantages from Cloud Computing technology, which are high computational power, scalability of the computational resources, easy access and pay-per-usage model. The results achieved prove Cloud Computing technology to be one of the most promising solutions to solve finally the long-lived problem of truly introducing the MC algorithms in the daily routine of the RT planning process.

## Abbreviations

MC: Monte Carlo
PHSP: Phase-space
PTV: Planning target volume
RT: Radiotherapy
SUT: Start-up time
TPS: Treatment planning system
VM: Virtual machine
